# Selections and Abstracts

**Published:** 1899-12

**Authors:** 


### SELECTIONS AND ABSTRACTS.

---

### SUCCESS IN THE PRACTICE OF MEDICINE.

The following letter of Dr. Herbert J. Hopkins, of Pittsburg, we copy from the *Philadelphia Medical Journal* of October 28 :

"There is and has been a great deal of talk about the so-called hospital and dispensary abuse; the general public though are quite satisfied, and rightly so, too, for none question the great good that such institutions are to the people at large, a wonderful dispensation of help and comfort to suffering humanity, poor and rich alike sharing the benefits. If an ambitious minority of the profession choose, for reasons of their own, to devote a large proportion of their waking hours to gratuitous work, the public are not to be blamed for availing themselves of such advantages. Of course it is becoming more difficult for practicing physicians of all kinds to compete with this free labor, but let the blame be placed anywhere but on the public who rightfully enjoy the good things that so rarely come their way.

"Charitable institutions are a distinguishing feature of Christian civilization—the keystone of Christianity being love or charity, which in a tangible form appears as a relief and comfort to the poor, suffering, and friendless. Philanthropists seeking the great returns find no field comparable to the establishment of hospitals, yet the promoters of hospitals find it impossible to keep them up on mere charitable donations, and rely largely for their support on the profits from pay-patients whose patronage is eagerly solicited; so that, while availing themselves of the free medical services provided by the institution, these same pay-patients are themselves furthering the good work.

"Through mismanagement or corruption it is possible that public charitable institutions may cease to fulfil the object for which they were originated, the gifts not reaching those for whom they were intended, in a proper way—an abuse indeed, but one not chargeable, as shown above, to the pay-patient.

"It would indeed appear unfair that the medical profession should not only render gratuitously all the medical services needed by those for whom the institution was founded, but should, in addition, contribute so laigely, if indirectly, to their proper support while there resident. As, long, however, as appointments on the various staffs are sought with such incredible avidity, there would not appear to be any real cause for alarm from that quarter. It is hard though for the general body of the profession, hard for the average physician, to achieve success, to be busy, and do the greatest possible amount of good honest work and gain a fair competence for his declining years, in the face of so much work done without a fee. However well drilled in the science of medicine, the modern physician, to achieve success, must needs be a master of that which constitutes the main reliance of the homeopathists, viz., the "art" of medicine. A working mastery of this is attained by nearly all practitioners in the course of years of experience. To the few it comes naturally, a gift as it were, and when seen in a state of perfection cannot but excite wonder and admiration, as illustrated in part by the common observation: 'Doctor ——— gets more credit out of a death than others do from a most brilliant recovery.' To be associated intimately with such a one for a year or two would be invaluable to the recent graduate. The old system of apprenticeship, though forever displaced by the different universities, had advantages which the latter can never supply.

"An important point is to make one's work acceptable to the patient, as thorough and effective as possible, and at the same time agreeable and pleasing. To do that it is almost indispenable to take a genuine and deep interest in each patient. Many complaints and disorders dismissed as trivial and unimportant by the busy physician are not so classified by the sufferers themselves, as the vast amount of money spent on the patent cure-alls testifies, and as frequently does the subsequent issue.

"Having once practiced general medicine, I can compare the work done by the general physician with that of the specialist, particularly that on the eye, ear, nose, and throat. The great difference is in the importance attached to the details. A specialist might truthfully be described as one who makes a special study of details—minutiæ overlooked or neglected by the physician. It is well to remember that while the field is broad, and it is impossible for one to know all that is to be known in all parts of it, the patients are comparatively few, and that the adversaries are free public services and patent medicines—adversaries well qualified to cause uneasiness to the great body of our beloved profession.

"In addition to making one's work thorough and effective as well as pleasing and agreeable, an important feature of the art is to give big value for money received, throwing into the scale time, interest, and sympathy.

Sectarians and semi-quacks are accused of playing 'the gentlemanly snare and sentimental game' to cover their ignorance. Any means so patent as to do that surely merits attention. It behooves us to be the real thing, and look it, gentlemen, in every sense of the word, confident, suave, and sympathetic. Though it is not given to all of us to be naturally magnetic, to easily attract and retain friends and supporters, we can all deserve and win them in abundance by being ideal physicians."—*West. Medical Review.*

---

### THE INCREASE OF CANCER.

Numerous writers have called attention to the constant increase in the frequency of the occurrence of carcinoma. The latest study of this subject, that of Joseph Frank Payne (*Lancet,* September 16, 1899), confirms the conclusions of former writers.

Cancer and tubercle are, beyond doubt, two of the most important subjects before the profession to-day. The former occurs with alarming frequency, and is steadily on the increase; the latter carries off one-seventh of our total population. Both have so far absolutely resisted all attempts that have been made to effect a cure. It is true that, in the case of cancer, the earlier and more thorough operations of to-day have brought about a slightly larger percentage of cures, or what are termed cures, and in other cases, no doubt, the interval between the operation and the recurrence has been prolonged. In this connection, it is to be understood that a person who has had a cancer removed cannot be said to be cured until many years have passed without a return of the disease. Some improvement has also been made in the treatment of tuberculosis chiefly in the lines of improving the patient's surrounding. If smallpox, typhus fever and other similar diseases can be stamped out or kept under control, there is every reason to hope that these two may also yield up the mysteries that have surrounded them—the influences that are responsible for their appearance and the means by which they may be cured or kept in check. Although the incalculable amount of work so far spent in these lines with but little result might tend to lessen the enthusiasm of future workers, there is every reason to continue to pursue the investigations and to believe that finally the reward will come.

It may be remarked, in passing, that Dr. Payne thinks the parasitic theory of cancer is decidedly less promising now than it was two years ago, although he does not deny such a possibility. His investigations have led him to the following "personal opinions" as to what is probable: "First," he believes that "there is an inherited bias or proclivity towards cancer in certain families; secondly, that the cases of supposed direct contagion are so exceedingly rare as to be probably due to coincidence; thirdly, indirect contagion cannot be proved, and in cases where it appears probable, the real explanation may be the separate action of the same local cause on the persons affected; and, fourthly, the operation of some external cause of infection is rendered probable by certain facts of geographical and local distribution." Among other interesting observations brought out are that the rate of increase of cancer in England is more rapid in males than in females, and the probability that cancer is relatively more common in the upper classes of society than in the lower. A table showing the death-rate from cancer in England for each decade since 1851 shows an increase in the last or fourth period of 86 per cent. for all ages, and of over 100 per cent. after the fifty-fifth year of life. In Scotland the mortality rate for carcinoma has steadily risen from 404 per 1,000,000 living persons, in 1861, to 770 in 1897. It is higher in the eight large towns of Scotland than in the "insular rural districts." In Ireland the death-rate is lower than in either England or Scotland. It shows an increase, however, from 370, in 1881, to 580 in 1897. In Norway the rate, in 1877, was 320, and in 1887 it was 600. In Prussia it was 310 in 1881 and 380 in 1887. In New York City the returns show a rise from 400, in 1875, to 530 in 1885. In New Zealand a steady increase has been noted since 1881.

The first criticism that would suggest itself in considering these figures is that the difference may be explained by more accurate diagnosis and by greater care in registration in recent years.

In reply to the first of these, Dr. Payne very justly says that there has not been any revolutionary advance in the matter of diagnosis of carcinoma and of sarcoma, for his figures include both; and while there is doubtless greater discrimination in the matter of registration, all authorities agree that this cannot explain the very great difference in the figures. In order that this source of error might be excluded as largely as possible, the present inquiry begins with the year 1851, before which the records are less reliable than since that time.

In the report of the Registrar-General of England for 1889, Dr. Ogle published a table showing the increase of cancer in different parts of the body from 1868 to 1888, which, even if not strictly accurate, is most instructive, at least. The largest increase was noted in cancer of the digestive organs, which amounted to 74 per cent. for males and 98 per cent. for females. Cancer of the tongue increased by 67 per cent. in men; cancer of the breast in women showed an increase of but 9 per cent., and of the uterine organs of 19 per cent. The general death-rate in this table had risen 50 per cent.

In Ireland, where the population is nearly stationary, the annual number of deaths from cancer, in 10 years has increased 25 per cent., and the death-rate 31 per cent. From the breast, the figures are 22 and 27 per cent. The deaths from cancer of the tongue have increased 40 per cent. in the same period. The records of St. Thomas's Hospital show that the admissions for cancer of the tongue have increased 83 per cent. in twenty years. From these and other figures it is estimated that the occurrence of cancer in this situation is increasing nearly 4 per cent. per annum.

Dr. Payne draws the following general conclusions from the facts presented: (1) Cancer is becoming increasingly frequent as a cause of death. (2) In this increase there is a marked predominance of cancer of the digestive organs. (3) Cancer of the generative organs increases less rapidly, but does not stand still.—*University Medical Magazine.*

---

### CERTAIN ASPECTS OF BONE AND JOINT DISEASE OF INTEREST TO THE GENERAL PRACTITIONER.

Virgil P. Gibney (*Medical News*, October 28, 1899) says: The injuries that commonly end in disease are not numerous, yet it would be well to bear in mind that trivial injuries are sometimes followed by most distressing consequences; that the term disease, as applied to these consequences, is not inappropriate. A slight wrench sometimes given by the mother or nurse, or a playmate, is followed so closely by symptoms that point unmistakably to disease of the bone that we are bound to recognize these traumata as factors at least in etiology. As a rule, these injuries are trifling and no lesion follows. Occasionally the hyperemia set up in the neighborhood of the joint furnishes a fertile soil for the bacilli which may have been introduced, long prior to this time, into the system, and a tubercular osteitis is the result.

Children convalescing from exanthemata or other infectious diseases are notoriously vulnerable, and the most trivial accidents are to be avoided. Curiously enough, the more severe traumata, such as falling from the roof or out of a window at the height of two or three stories, or from runaway carriages, seldom end in disease. The result of these severe injuries is immediate. Again, in adult life more especially, bruises or frequently recurring blows in the same locality end in sarcomata or other malignant growths. For a study of this subject reference is made to the Annals of Surgery, March, 1898, article written by Dr. Colby. He urges an early diagnosis of neoplasms in order that the spread of the process may be checked and radical measures instituted in order to prolong the life of the patient. Repeated blows sometimes impair the walls of the blood-vessels and rupture takes place, producing a hematoma in the deeper structures which may be mistaken for neoplasm. Aspiration or incision will clear the diagnosis.

It may be stated, with little fear of contradiction, that slight injuries occurring in a patient whose ancestry is rheumatic or gouty in a fair percentage of instances bring out or develop rheumatism or gout and a general rheumatoid disease is developed. He cites an instance in which a gentleman near fifty years of age fractured the dorsal ligament connecting the second and third phalanges; prompt measures of relief were adopted, and yet a general breaking up of the system or a general development of rheumatic or gouty lesions followed in other joints. Take subacute rheumatic arthritis of the knee, for instance. This is frequently induced by slight trauma and acute synovitis follows almost immediately. Resolution seems to be established, and yet the recovery is not complete. There are three diseases that are most persistent and impair the functions of the joints frequently—tuberculosis, sarcoma and rheumatism.

There are certain diseases that naturally end in deformity, adopt what treatment we may—rickets, poliomyelitis, tuberculous osteitis, rheumatism, gout and neoplasms involving the epythisis. Rickets is followed by pigeon breast, scoliosis, kyphosis, coxa vara, and deformities of the pelvis and extremities. Orthopedic surgeons are often called upon to correct these deformities. When the cause is known he may frequently dispense with apparatus in so doing.

*Poliomyelitis:* Clinical evidence indicates possibility of infectious nature of this disease. It is a very difficult disease to manage. The spinal column, hip, knee, ankle and shoulder, especially, present deformities that appliances and operations of all kinds sometimes fail to correct. Joints may be protected and muscles may be transplanted; capsular ligaments may be shortened and even joints may be removed, substituting therefor a synostosis, which enables the patient to go about with more comfort, yet leaving some disability. Brilliant work has been accomplished by surgeons in the relief of paralytic deformities. A careful diagnosis must be made of the particular muscles involved and those which will be of the most assistance to the member after transplantation. Special effort should be made to limit the disease in the chord as soon as possible. Protection should be thrown about the muscles and joints and early use should be prevented.

Rheumatism does not often deform the child, but it is a possibility. More frequently it does deform adults, and is exceedingly difficult to correct in any age. Our efforts, therefore, should be all the more vigorous in preventing such disabilities.

Empyema may produce most distressing curvatures of the spine.

*Spinal Injuries:* Strains, concussions, bruises in children and adults may occasionally end in disease. They are to be differentiated from Pott's disease, etc. Some of the points in differential diagnosis between disease and injury are the gait, the stiffness of the spinal column, impairment of function and location of pain, whether in the course of certain nerves or whether localized. In investigating spinal disease, the absence of tenderness along the spine should not exclude the possibility of such disease. In vertebral disease, involving the bodies of the vertebræ, tenderness is the exception, especially in children. The nerve passing through the foramina of exit does not supply, as a rule, the spinous process of the vertebra involved. Spinal tenderness is the rule in adolescence in the female, even when much deformity exists, and when one feels positive that tubercular lesion is present. The occurrence of irritable spine in young women, and even in old women, should prompt one to examine the spine in regard to its function. Insist upon putting the spinal column through all the different movements possible to it. Have them come down sharply upon the heels. Produce sudden pressure upon the head. Flex the thighs strongly upon the abdomen, etc. Radiating pains in the course of the nerves passing from the different regions are very significant, especially girdle-like sensation about the abdomen; if in the cervical region, the occipital pains; in the lumbo-sacral region, the symmetrical sciatic pains.—*Chicago Clinic.*

---

THE SIGNIFICANCE OF OBSTINATE CONSTIPATION AS A SYMPTOM.

Dr. J. G. Sherrill (*The Louisville Medical Monthly*, November, 1899) discusses the subject of obstinate constipation in those cases where there is a complete cessation of gas or fecal flow, or an inability to produce evacuation of either. He says the condition is frequent, and may be gradual in development, the evacuations being more and more difficult until absolute constipation is etablished.

This variety occurs most frequently in women of sedentary habits, who do not heed the calls of nature. The gradual encroachment upon the lumen of the gut by a neoplasm is also a frequent cause. Among the causes of sudden obstipation he cites: Simple impaction of feces; the practice of toxins in the alimentary canal, as in the condition known as biliousness, where long and continued medication is necessary to produce an evacuation; peritonitis, where there is paresis of the intestine or that form known as the septic type. Volvulus, intussusception, strangulated hernia, and intestinal bands, all produce this symptom mechanically at first, later by the paresis incident to the peritonitis. He asserts that laparotomy for simple constipation would be a serious mistake, but not nearly so grave as to fail to recognize a mechanical obstruction in which an operation would offer the only hope for the preservation of life. He urges that, inasmuch as the treatment depends so much upon the cause, our methods of diagnosis should, if possible, be improved upon. He states that simple constipation will seldom be accompanied by such severe pain as results from a mechanical obstruction. Fecal vomiting occurs late, when an impaction is present.

Lead poisoning can be recognized by the paroxysmal character of the pain, which is received by pressure, absence of fever, retraction and rigidity of abdominal walls, and blue line on the gums, if present, is a positive sign.

Tumors and malignant diseases can usually be recognized by physical examination, also by the slow and gradual development of the symptoms.

Intussusception will often present a soft sausage-shaped tumor, and usually the constipation will not be complete.

Strangulated hernia and volvulus are marked by pain, vomiting (which is soon fecal), rapid distention of abdomen, low temperature and anxious countenance. Auscultation will reveal contraction of intestines above constriction.

Peritonitis is accompanied by fever of at least one or two degrees. Pain is not always present, and may or may not be severe. Pressure increases it. Stethoscope will reveal no contraction of the intestines. Peritonitis is due to rupture or wound of the intestine, there will probably be a tympanitic note over normal liver dullness, owing to the free gas in the peritoneal cavity.

All the conditions producing obstinate constipation have many features in common, and on that account demand a more careful attention to the minor points for a diagnosis. The facial expression is often indicative of the gravity of the case.—*Chicago Clinic.*

---

### THE CHARACTER OF THE AUTHOR OF "SCIENCE AND HEALTH."

To the mentally balanced individual there are few things so calculated to arouse astonishment as the establishment and spread of so-called Christian Science, and the influence wielded over so many persons of seemingly average intelligence and common sense by its priestess, Mrs. Mary B. G. Eddy. Meriting as it does, neither the name of religion nor of science, and utterly unreasonable in the absurd conglomeration of meaningless terms in which its extravagant claims are set forth, it has nevertheless become the fetish before which hundreds of deluded victims bow. Its spread among a civilized and cultured people is only another proof that human nature is ever the same, in the first as in the twentieth century; in the person of an educated American, and that of the ignorant savage.

We note a recent account in the lay press of a libel suit brought by a former disciple of Mrs. Eddy for one hundred and fifty thousand dollars. It is set forth in the complaint among other things that this priestess of humanity not only claims to restore the sick and awaken the dead (for a money consideration)

but that she from "motives of retribution and revenge," employs her alleged mental powers to produce sickness and death among those of her followers, or their families, who have waxed rebellious to her authority. It appears then that the author of "Science and Health" is not an unmixed blessing to humanity. The most surprising part is not the extraordinary claims made by this colossal egotist, but that at this period of the world's history there should be people who sincerely believe this sort of thing. We of the South are familiar with beliefs of this kind among the more ignorant class of negroes, but had not supposed that such an anachorism could find a congenial atmosphere in the vicinity of cultured Boston.

A Massachusetts paper has recently published the result of an investigation of the private character of Mrs. Eddy, which is doubtless a surprise to many people who have hitherto regarded her as a kindly fanatic. The article in question shows that this priestess of humanity is not only the possessor of great wealth, made out of the susceptible individuals who stand open-mouthed to swallow her stories, but is so vindictive and venomous in her hatred as to arouse the most superstitious dread, of her supposed occult powers, in the mind of her disciples. The newspaper conducting the investigation claims to have legal proof that instead of being in the robust health which is her boast, Mrs. Eddy is in reality a physical wreck, and is herself a victim of the morphine habit. A distinguished writer says that all the "mind sects except Christian Science have lucid intervals, in which they confess that they are not the equals of the diety."—*North Carolina Medical Journal.*

---

### POINTS IN THE ARSENICAL CAUSTIC TREATMENT OF CUTANEOUS CANCERS.

1. The arsenious acid caustic treatment of skin cancers does not contemplate or depend upon the actual destruction of the new growth by the caustic.

2. The method is based upon the fact that newly formed tissue of all kinds has less resisting power than the normal structure when exposed to an irritation and its consequent inflammation. Hence the former breaks down under an "insult" which the latter successfully resists.

3. If, therefore, the whole affected area can be subjected to the influence of an irritant of just sufficient strength to cause a reactive inflammation intense enough to destroy the vitality of the new cells, the older normal cells will survive.

4. Arsenious acid of properly mitigated strength is such an agent, and its application causes an inflammation of the required intensity.

5. It, therefore, exercises a selective influence upon the tissues to which it is applied, and causes the death of the cancer cells in localities outside the apparent limits of the new growth, where there is as yet no evidence of disease.

6. It is superior, in suitable cases, to any method, knife or cautery, which requires the exercise of the surgeon's judgment as to the extent to which it is to be carried. That the judgment is often wrong, and necessarily so, is shown by the frequency of recurrence under these methods even in the best hands.

7. It is applicable to all cutaneous carcinomata in which the deeper structures are not involved and which do not extend far on to the mucous membranes.

8. It is easy of application, it is safe, it is only moderately painful and its results compare favorably with those obtained by other methods.—*William S. Gottheil, M.D.*

---

### THE LIFE INSURANCE EXAMINER.

Dr. Brandeth Symonds makes a plea in the *Medical Examiner* for undergraduate instruction in making life insurance examinations. There are probably about 200,000 examiners' positions in the United States and Canada, and these are occupied by about 50,000 physicians. The regular life insurance companies distribute to these men about $5,000,000 annually, and the money is in almost every case certain. Why then should not the examiners be trained for their work? They too often are not. They are young men who are too careful and drive away cases on account of their painstaking scrutiny of the whole body, or careless men who let cases slip through in order to keep in with the agent.

As a rule the examiner is appointed more on account of his influence with the agent than for his especial ability.

Dr. Symonds is authority for the statement that of all the medical schools in the United States and Canada but two give undergraduate instruction in life insurance examination. These are the Medical College of Virginia and the University of Minnesota at St. Paul. There may be a few others that give some kind of instruction in this branch, but these two are the only schools that pretend to give and do actually give good courses. Such a course, says Dr. Symonds, should cover at least the following topics:

1. Some instruction in vital statistics and the fundamentals of life insurance. 2. The relations of the examiner to the company and the applicant. 3. The facts concerning each disease, which are of importance from a life insurance point of view. 4. Habits, occupation and environment. 5. The family record and hereditary. 6. The physical examination, particularly with reference to the distinction between essentials and non-essentials. 7. The relation of the examiner to the agent. 8. Frauds and fraudulent practices.

The careless appointment of poor men has too often put a slur on the examiner, and some agents have little respect for the examiner and his work.—*Maryland Medical Journal.*

---

### CRAIG COLONY PRIZE FOR ORIGINAL RESEARCH IN EPILEPSY.

Last year Dr. Frederick Peterson, President of the Board of Managers of the Craig Colony for Epileptics, offered a prize of $100.00 for the best original contribution to the Pathology and Treatment of Epilepsy.

The seven papers received were submitted to three members of the New York Neurological Society, who gave to the Board of Managers of the Craig Colony the following report:

NEW YORK, October 10, 1899.

*To the Board of Managers, Craig Colony, Sonyea, N. Y.:*

SIRS:—The Committee on the Craig Colony Prize for Original Work in Epilepsy has decided that no award should be made this year. Some of the essays submitted failed to comply with the conditions of the competition, others were more limited in scope than a successful essay should be. Three deserve special mention. Their titles and devices are:

"The Pathology of Epilepsy," by Co. $\left\{ \begin{array}{l} NH_2 \\ OH,NH_3 \end{array} \right.$

"Status Epilepticus," by "Aura," and

"The Pathology of Epilepsy," by "On and Ever Onward."

Respectfully, PEARCE BAILEY,
Secretary N. Y. Neurological Society.
GEO. W. JACOBY,
Ex-President N. Y. Neurological Society.
IRA VAN GIESON,
Director Pathological Inst. of the N. Y. State Hospitals.

The prize of 1899 not having been awarded in accordance with the report of the Committee, Dr. Peterson now offers a prize of $200.00 for the year 1900 under similar conditions. This sum will be awarded to the author of the best contribution to the Pathology and Treatment of Epilepsy. Originality is the main condition.

The prize is open to universal competition, but all manuscripts must be submitted in English. Each essay must be accompanied by a sealed envelope containing the name and address of the author and bearing upon the outside a motto or device, which is to be inscribed upon the essay.

All papers will be submitted to a similar committee, consisting of three members of the New York Neurological Society, and the award will be made upon its recommendation at the annual meeting of the Board of Managers of the Craig Colony October 9th, 1900.

Manuscripts should be sent to Dr. Frederick Peterson, No. 4 West Fiftieth street, New York City, on or before September 1st, 1900. The successful essay becomes the property of the Craig Colony and will be published in its medical report.

---

### CONSERVATION IN PELVIC SURGERY.

The following rules may be laid down for guidance in the practice of conservative pelvic surgery:

1. An ovary should not be removed simply because the tube of its side has been taken out.

2. Wherever it is possible in cases of parovarian cyst, the ovary should be left.

3. In cases of adherent ovary, every effort should be made to so relieve its adhesion as to not tear it out from its insertion into the broad ligament, with a view of leaving it intact.

4. So-called Graafian cysts may be punctured with a knife or scissors, or by cautery.

5. Hematomata may often be dissected out where they do not involve too much of the ovarian tissue.

6. Frequently small ovarian cystomata and ovarian abscesses may be so manipulated as to preserve the functions of the ovary.

7. Tubes may be preserved not infrequently by proper care. Adherent tubes are released, strictured tubes are opened, and tubal abscesses may be frequently drained.—*Thomas B. Eastman, M.D., Indiana Medical Journal.*

---

### A MEDICAL DEFENCE UNION.

Not so very long ago a number of the profession in the city of Toronto were solicited in such a way that, if it became necessary in defending a certain action, or if damages were to be paid, each were to guarantee a payment in order to assist the professional brethren so mulcted. Suits for malpractice are every little while cropping up, and if the unfortunate one who finds himself a defender in a suit for damages in these cases, whether resting upon sufficient evidence or merely trumped-up charges, be a young practitioner who has not as yet reached that delightful stage where he is going forward instead of going behind in his annual receipts, it would indeed be a very comforting thought to him to know that he had at his back, come what may, an association that would defend his action and pay the damages if assessed against him. We are not aware that any such society has existence in this country, but we have no doubt that it could be made workable and meet with popular favor. Possibly a defence association could be organized in connection with any of our local or national medical associations, and, if need be, members, if they saw fit, could enroll themselves on the membership of both local and national associations of this character. Then the Medical Council might take this matter into their serious consideration and devise a scheme by which licentiates of that body would be protected from these very annoying charges, often putting a practitioner to no end of worry, trouble, and expense for exceedingly slight causes. There is no doubt that an insurance bureau of this character could be carried on by the Medical Council and prove of incalculable benefit to the profession at large. The annual cost to each individual who chose to take advantage of such an undertaking would probably not be large, and we think some steps ought to be immediately taken to institute such an association.—*Dominion Medical Monthly.*

---

### A NEW METHOD OF LOCAL ANESTHESIA IN OPERATIONS ON THE TYMPANIC MEMBRANE.

Borrain—*Laryngoscope.*—The author has obtained good results from the following formula:

| | | |
|---|---|---|
| ℞ | Phenol | 10 grs. |
| | Menthol | 5 grs. |
| | Chlorhydrate of cocain | 5 grs. |

Or equal parts of each may be employed. After cleansing and sterilizing the auditory canal, the anesthetizing agent is applied by means of a pledget of absorbent cotton. In the cases in which it was used there was at first a light burning sensation, which was followed by complete local anesthesia in three minutes.—*North Carolina Medical Journal.*

---

### DEATH IN CONSEQUENCE OF PROLONGED LAUGHTER.

Wachholz (*Przeglad lekarski*) relates the case of a peasant girl, eighteen years old, who was seized in a field by two fellows and tickled violently on the breast. She died as a result of laughter produced by the tickling. The author explains the occurrence of death from laughing as follows: Laughter consists of an inspiration followed by short and more or less deep expirations. In forcible expiration the abdominal muscles contract and compress the intestines and the diaphragm. Long-continued pressure on the diaphragm acts upon the vagus and diaphragmatic nerves, exciting them and finally paralyzing them. We are not convinced that this is an altogether satisfactory explanation. Certainly the mechanism of death from laughing, if it ever occurs in a healthy person, is worthy to be studied by the physiologists.—*New York Medical Journal.*

---

### THE DEATH-DEALING LONG-TUBE NURSING BOTTLE.

The object of this paper is briefly to give the perilous and untrustworthy features of the so-called long-tube nursing bottle, and to show justification for not only interdicting it, but doing so by law. The peculiar features of danger from its employment may be summarily stated, that owing to its construction and material and to the use to which it is put, probably no better incubator of pernicious micro-organisms and their toxins could be easily designed, as none have proven so successful. A series of investigations was made, microscopically, bacteriologically and chemically, establishing beyond question the danger of these tubes for the introduction of pathogenic microbes into the infantile economy.—*E. Wende, Med. Rev.*

---

### CURE FOR OPIUM HABIT.

The opium habit prevails among Europeans in the East to an extent which is appreciated only by those who are brought into contact with its victims. In view of this fact the announcement by McLeod, of Shanghai, that it is possible to cure the habit by the administration of sodium bromide, is indeed welcome news. He gives the drug in two doses of two drachms, in solution, every two hours for the first two days, and one drachm on the third day. Two ounces in all will probably suffice in most cases.—*Medical Times.*

---

### SYPHILITIC IRITIS.

The chief symptoms are (1) ciliary congestion with a certain amount of pain referred to the forehead or temple, photophobia, and lacrymation; (2) discolored iris, sluggish action of the pupil, which dilates irregularly under atropine; (3) adhesions to the lens, deposit of uveal pigment, nodules of lymph in or on the iris.—*Jonathan Hutchinson, Jr., Medical Record.*

